# Serotonin: a platelet hormone modulating cardiovascular disease

**DOI:** 10.1007/s11239-020-02331-0

**Published:** 2020-11-05

**Authors:** Marina Rieder, Nadine Gauchel, Christoph Bode, Daniel Duerschmied

**Affiliations:** 1 Department of Cardiology and Angiology I, Faculty of Medicine, Heart Center Freiburg University, University of Freiburg, Hugstetter Strasse 55, 79106 Freiburg, Germany; 2 Department of Medicine III (Interdisciplinary Medical Intensive Care), Medical Center, Faculty of Medicine, University of Freiburg, Freiburg, Germany

**Keywords:** Serotonin, 5-hydroxytryptamin, Platelets, Cardiovascular disease

## Abstract

Cardiovascular diseases and depression are significant health burdens and increasing evidence suggests a causal relationship between them. The incidence of depression among patients suffering from cardiovascular disease is markedly elevated, and depression itself is an established cardiovascular risk factor. Serotonin 5-hydroxytryptamin (5-HT), a biogenic amine acting as a neurotransmitter and a peripheral hormone, is involved in the pathogenesis of both, cardiovascular disease and depression. Novel cardiovascular functions of 5-HT have recently been described and will be summarized in this review. 5-HT has a broad spectrum of functions in the cardiovascular system, yet the clinical or experimental data are partly conflicting. There is further research needed to characterize the clinical effects of 5-HT in particular tissues to enable targeted pharmacological therapies.

## Highlights

- Depression and cardiovascular diseases are significant health burdens.
- Serotonin, acting as a neurotransmitter and a biogenic amine is involved in the pathogenesis of depression and cardiovascular disease.
- Novel cardiovascular functions of serotonin have recently been described.
- This review focuses on the role of serotonin in atherosclerosis, myocardial infarction, heart failure, thrombosis and arterial hypertension.

## Serotonin

Serotonin 5-hydroxytryptamin (5-HT) was discovered more than 70 years ago and first described as a vasoconstrictor [1]. Since then, multiple functions of 5-HT emerged, all conducted via signaling through one of the so far 15 known distinct 5-HT receptors [2, 3] or by covalent binding to different effector proteins, named “serotonylation” [4]. In regard of cardiovascular diseases, the receptor subtypes 5-HT1B, 5-HT2A, 5-HT2B, 5-HT4 and 5-HT7 are of particular interest. 5-HT1B, 5-HT2A, 5-HT2B and 5-HT7 are expressed on smooth muscle and endothelial cells of arteries and veins, regulating vascular tone. 5-HT2A is additionally located on platelets and involved in activation and aggregation, and it can also be found on cardiomyocytes and fibroblasts. 5-HT4, expressed in cardiac atria and ventricle conducts positive inotropic and lusitropic effects but may also trigger arrhythmias (reviewed in [5]).

## Serotonin synthesis

5-HT is derived from the essential amino acid l-tryptophan [6]. The biosynthesis of 5-HT is regulated by two isoforms of the enzyme tryptophanhydroxylase (Tph), Tph1 and Tph2 [7]. Tph2 is expressed in the brain stem, where it regulates 5-HT synthesis in the central nervous system [8]. The effects mediated by central 5-HT are very complex: It is involved in the regulation of mood [9], appetite [9], circadian rhythm [10] and sexual drive. Disturbances in this system appear to be closely linked to psychiatric diseases like depressive or anxiety disorders [11].

**Fig. 1:**
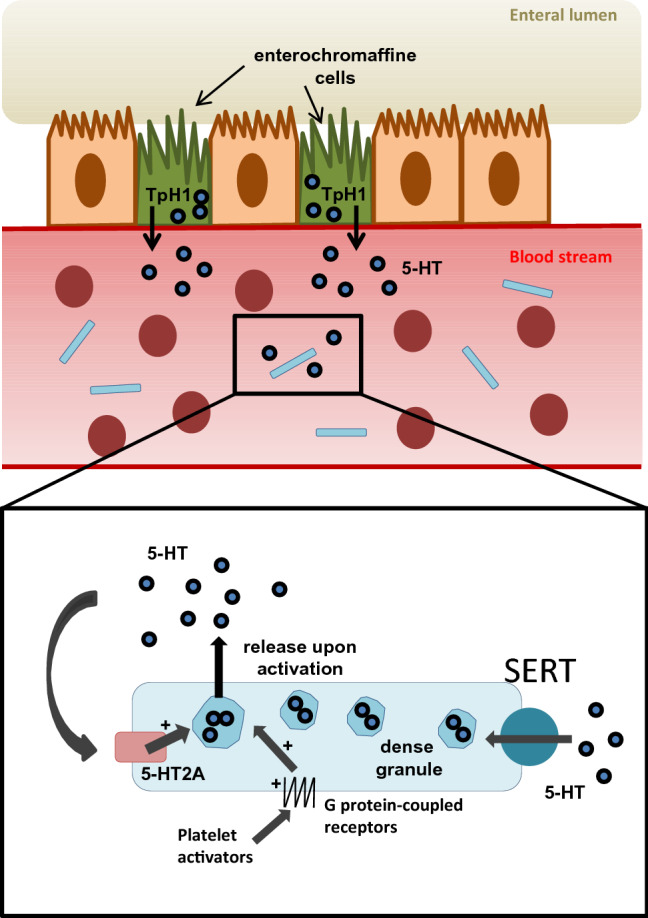
Enterochromaffine cells (EC-cells) in the gut express Tph1 and synthesize the majority of peripheral serotonin (5-HT). EC-cells release 5-HT into the blood plasma, where it is taken up by platelets (blue rectangles) via SERT. Platelets, as the main circulating reservoir, store 5-HT in their dense granules. Upon activation, platelet dense granules release 5-HT. 5-HT can amplify the release of dense granules via activation of the 5-HT2A receptor

However, the vast majority of 5-HT can be found in the peripheral system [12]. Peripheral 5-HT is synthesized by enterochromaffine cells in the gut by tryptophan hydroxylase I and released into blood plasma [13]. Most of the circulating 5-HT is taken up by platelets via the 5-HT transporter SERT [14]. Platelets, as the main circulating reservoir of 5-HT, store it in their dense granules in high concentrations and release it upon activation [4]. As platelets are not able to synthesize 5-HT, chronic intake of selective serotonin reuptake inhibitors (SSRIs) and therefore long-term blockage of SERT results in a depletion of platelet 5-HT storage [15] (Fig. 1).

In peripheral tissues, 5-HT is involved in a broad variety of functions including regulation of vascular tone [16], gut motility [17], hemostasis [18], and immune responses [19–21]. Due to the above described broad distribution of receptors in the cardiovascular system, 5-HT plays a major role in a variety of cardiovascular diseases and elevated 5-HT concentrations have been described in e.g. arterial hypertension [22], carotid atherosclerosis [23] or coronary artery disease [24, 25].

## Serotonin in hemostasis and thrombosis

Platelets play a major role in hemostasis and thrombus formation. 5-HT influences platelet activation and aggregation by enhancing multiple pathways of primary hemostasis: Primary hemostasis is induced by circulating von Willebrand-Factor (vWF) binding to exposed collagen at sites of vascular endothelial damage. Platelets are able to adhere on vWF via glycoprotein (GP) Iβα expressed on their surface [26]. This stable binding allows the interaction of collagen with GP IIb/IIIa on platelets, leading to platelet activation [27]. Upon activation, a number of intracellular signaling mechanisms, mostly G-protein-dependent, lead to an exocytosis of dense granules. These release a broad variety of molecules, such as ADP, ATP, Ca^2+^ and 5-HT. As platelets themselves express corresponding receptors for these released substances including 5-HT receptor 2A and 3, dense granule secretion leads to a feedback loop enhancing platelet aggregation and activation at the site of vascular damage (reviewed in [28]). Additionally, serotonin is taken up in the cytoplasm and transamidated to small GTPases by transglutaminases during activation and aggregation of platelets, a process called serotonylation [29]. This triggers the further release of dense granules [29]. Another mechanism how 5-HT influences platelet activation and aggregation is by altering N-glycan expression on the platelet surface [30]. When the serotonin transporter is lacking, an agonist-induced Ca^2+^ influx through store operated Ca2 + entry (SOCE), integrin activation, degranulation and aggregation responses to glycoprotein VI and C-type lectin like receptor 2 (CLEC-2) is reduced in platelets [31].

The importance of 5-HT in the process of platelet activation and aggregation was shown in animal models: 5-HT infused mice suffer from enhanced platelet aggregation [30, 32, 33]. This can be normalized by the intake of SSRI [30, 32] or a 5-HT2a receptor antagonist [30, 33]. Tph1 deficient mice with low peripheral 5-HT levels exhibit a mildly prolonged bleeding time due to an impaired release of dense granules. This results in a reduced risk of arterial thrombosis and experimental venous thromboembolism [29]. In an in vivo dog model of spontaneous occlusive coronary thrombus formation, increased plasma serotonin levels could be measured during thrombus formation [34]. This could be reproduced in a model of carotid artery thrombosis in rats, where a 15 fold increase of 5-HT was measured [35].

In humans, SERT-levels are associated with venous thrombosis [36] and in patients suffering from carcinoid syndrome, elevated 5-HT levels are associated with a hypercoagulable status [28]. Epidemiologic data suggests a role of 5HT or 5HT-reuptake inhibitors in the development of venous thrombosis. Patients with depression were reported to have higher incidences of venous thromboembolism in general and the use of tricyclic antidepressants, selective serotonin reuptake inhibitors and other antidepressants were each associated with an increased VTE risk [37] The mechanisms behind the role of serotonin in venous thrombosis have not been addressed so far.

## Serotonin in atherosclerosis

Clinical studies on cardiovascular effects of depletion of platelet 5-HT by intake of SSRIs are inconclusive. On the one hand, some did not find any increase or even an ameliorated cardiovascular risk [38–41], whereas others reported a worse cardiovascular risk profile after SSRI intake [42–44].

Blockage of the 5-HT2A receptor by the antagonist sarpogrelate retards the progression of atherosclerosis in a rabbit model by upregulation of eNOS and presumably antiproliferative effects on smooth muscle cells and macrophages [45]. Consistent with these findings, sarpogrelate has a therapeutic effect an patients with atherosclerosis obliterans [46] and critical limb ischemia [47]. In a collective of diabetic patients, sarpogrelate decreased coronary artery plaque volume [48].

Consistent with that, animal studies showed that chronic intake of Fluoxetine enhances atherosclerosis by promoting myeloid binding capacity and increasing leukocyte-endothelial interactions [49].

## The role of serotonin in myocardial infarction

Depletion of platelet serotonin storages by long-term administration of serotonin reuptake inhibitors reduces the risk of MI [50–52]. Consistent with that, a previous study revealed that possessing the LL genotype of SERT resulting in a higher expression and activity of SERT leads to a significantly increased risk for MI [53]. Moreover, serotonin levels are associated with coronary artery disease and occurrence of cardiac events [24]. Mechanistically, serotonin is thought to promote these adverse effects by enhancing platelet aggregation and vasoconstriction of diseased coronary arteries (reviewed in [54]).

Myocardial infarction leads to platelet activation, subsequently resulting in a further release of 5-HT from platelets, which in turn worsens myocardial ischemia and promotes reperfusion injury [55]. Recently, our group showed that the 5-HT-mediated aggravation of reperfusion injury is due to enhanced neutrophil degranulation leading to enhanced inflammation in the infarct area [15].

Animal studies on pharmacological blockage of 5-HT or its receptors led to conflicting results, mainly due to a broad variety in experimental setups, animal models and serotonin receptor antagonists used. The 5-HT2 receptor antagonist LY53857 did not influence infarct size in a canine model of myocardial infarction [56]. Contrary to these in vivo results, it was reported that LY53857 increased reperfusion injury ex vivo [57], whereas it was reduced by several other 5-HT2 receptor antagonists [58]. The combined Ca2 + and 5-HT2 receptor antagonist neopamil reduced infarction size in pigs [59] and the 5-HT2A receptor antagonist sarpogrelate did the same in rabbits by inhibiting 5-HT release [55].

## Serotonin in heart failure

The 5-HT4 receptor is expressed in atria and ventricles at a very low level under physiologic conditions. In heart failure, the expression of 5-HT4 is markedly upregulated, and stimulation of 5-HT4 receptor increases myocardial contractility and relaxation [60]. Overall, the mechanisms of action resembles that from beta-adrenoceptors through a pathway involving cAMP and PKA-mediated phosphorylation of proteins of Ca^2+^ handling, resulting in enhanced contractility through increased Ca^2+^ availability [61, 62]. But as the increased contractility via cAMP is energy-intensive, a blockage of this pathway e.g. by beta-adrenoceptor antagonists is beneficial in heart failure patients. It was thought that a blockage of 5-HT4 could be beneficial in the same way [61]. Yet treatment of heart failure in rats with the 5-HT4 antagonist piboserid resulted in only small beneficial effects [63] and human studies were disappointing due to a high number of adverse events [64].

## Serotonin and hypertension

Elevated 5-HT levels have been reported for patients with arterial hypertension [65, 66] and these have an altered platelet surface profile [67].

Peripheral administration of 5-HT leads to a triphasic response of blood pressure: Due to the stimulation of 5-HT3 receptors on vagal afferents, initially a short vasodepressive phase occurs. Then, activation of 5-HT2A receptors leads to a vasopressive phase and finally, the activation of 5-HT7 receptors on smooth vascular muscle cells leads to another vasodepression [28]. Central administration of 5-HT can cause hypertension via activation of 5-HT2 receptors or hypotension by stimulation of 5-HT1A receptors. The antihypertensive drug Urapidil acts via antagonism on central adrenoceptors but also via agonism on central 5-HT1A receptors [28].

## Concluding remarks

5-HT has a broad spectrum of functions in the cardiovascular system, yet the clinical or experimental data are partly conflicting. There is further research needed to characterize the clinical effects of 5-HT in particular tissues to enable targeted pharmacological therapies.

## References

[1] Rapport MM, Green AA, Page IH (1948). Serum vasoconstrictor, serotonin; isolation and characterization. J Biol Chem.

[2] McCorvy JD, Roth BL (2015). Structure and function of serotonin G protein-coupled receptors. Pharmacol Ther.

[3] David DJ, Gardier AM (2016). The pharmacological basis of the serotonin system: application to antidepressant response. L’Encephale.

[4] Walther DJ, Peter J-U, Winter S (2003). Serotonylation of small GTPases is a signal transduction pathway that triggers platelet alpha-granule release. Cell.

[5] Ayme-Dietrich E, Lawson R, Da-Silva S (2019). Serotonin contribution to cardiac valve degeneration: new insights for novel therapies?. Pharmacol Res.

[6] Bender DA (1983). Biochemistry of tryptophan in health and disease. Mol Aspects Med.

[7] Swami T, Weber HC (2018). Updates on the biology of serotonin and tryptophan hydroxylase. Curr Opin Endocrinol Diabetes Obes.

[8] Mohammad-Zadeh LF, Moses L, Gwaltney‐Brant SM (2008). Serotonin: a review. J Vet Pharmacol Ther.

[9] Strasser B, Gostner JM, Fuchs D (2016). Mood, food, and cognition: role of tryptophan and serotonin. Curr Opin Clin Nutr Metab Care.

[10] Morin LP (1999). Serotonin and the regulation of mammalian circadian rhythmicity. Ann Med.

[11] Żmudzka E, Sałaciak K, Sapa J, Pytka K (2018). Serotonin receptors in depression and anxiety: insights from animal studies. Life Sci.

[12] Veenstra-VanderWeele J, Anderson GM, Cook EH (2000). Pharmacogenetics and the serotonin system: initial studies and future directions. Eur J Pharmacol.

[13] Walther DJ, Peter J-U, Bashammakh S (2003). Synthesis of serotonin by a second tryptophan hydroxylase isoform. Science.

[14] Mercado CP, Kilic F (2010). Molecular mechanisms of SERT in platelets: regulation of plasma serotonin levels. Mol Interv.

[15] Mauler M, Herr N, Schoenichen C (2019). Platelet serotonin aggravates myocardial ischemia/reperfusion injury via neutrophil degranulation. Circulation.

[16] Myers JH, Mecca TE, Webb RC (1985). Direct and sensitizing effects of serotonin agonists and antagonists on vascular smooth muscle. J Cardiovasc Pharmacol.

[17] Sanger GJ (1996). 5-Hydroxytryptamine and functional bowel disorders. Neurogastroenterol Motil.

[18] Duerschmied D, Bode C (2009). The role of serotonin in haemostasis. Hamostaseologie.

[19] Shajib MS, Khan WI (2015). The role of serotonin and its receptors in activation of immune responses and inflammation. Acta Physiol Oxf Engl.

[20] Schoenichen C, Bode C, Duerschmied D (2019). Role of platelet serotonin in innate immune cell recruitment. Front Biosci Landmark Ed.

[21] Mauler M, Bode C, Duerschmied D (2016). Platelet serotonin modulates immune functions. Hamostaseologie.

[22] Frishman WH, Grewall P (2000). Serotonin and the heart. Ann Med.

[23] Ban Y, Watanabe T, Miyazaki A (2007). Impact of increased plasma serotonin levels and carotid atherosclerosis on vascular dementia. Atherosclerosis.

[24] Vikenes K, Farstad M, Nordrehaug JE (1999). Serotonin is associated with coronary artery disease and cardiac events. Circulation.

[25] van den Berg EK, Schmitz JM, Benedict CR (1989). Transcardiac serotonin concentration is increased in selected patients with limiting angina and complex coronary lesion morphology. Circulation.

[26] Ruggeri ZM, Mendolicchio GL (2007). Adhesion mechanisms in platelet function. Circ Res.

[27] Denis CV, Wagner DD (2007). Platelet adhesion receptors and their ligands in mouse models of thrombosis. Arterioscler Thromb Vasc Biol.

[28] Fraer M, Kilic F (2015). Serotonin: a different player in hypertension-associated thrombosis. Hypertens Dallas Tex.

[29] Walther DJ, Peter J-U, Winter S (2003). Serotonylation of small GTPases is a signal transduction pathway that triggers platelet alpha-granule release. Cell.

[30] Mercado CP, Quintero MV, Li Y (2013). A serotonin-induced N-glycan switch regulates platelet aggregation. Sci Rep.

[31] Wolf K, Braun A, Haining EJ (2016). Partially defective store operated calcium entry and hem(ITAM) signaling in platelets of serotonin transporter deficient mice. PLoS ONE.

[32] Ziu E, Mercado CP, Li Y (2012). Down-regulation of the serotonin transporter in hyperreactive platelets counteracts the pro-thrombotic effect of serotonin. J Mol Cell Cardiol.

[33] Przyklenk K, Frelinger AL, Linden MD (2010). Targeted inhibition of the serotonin 5HT2A receptor improves coronary patency in an in vivo model of recurrent thrombosis. J Thromb Haemost JTH.

[34] Benedict CR, Mathew B, Rex KA (1986). Correlation of plasma serotonin changes with platelet aggregation in an in vivo dog model of spontaneous occlusive coronary thrombus formation. Circ Res.

[35] Wester P, Dietrich WD, Prado R (1992). Serotonin release into plasma during common carotid artery thrombosis in rats. Stroke.

[36] Llobet D, Vallvé C, Tirado I (2019). VAMP8 and serotonin transporter levels are associated with venous thrombosis risk in a Spanish female population. Results from the RETROVE Project. Thromb Res.

[37] Parkin L, Balkwill A, Sweetland S (2017). Antidepressants, depression, and venous thromboembolism risk: large prospective study of UK women. J Am Heart Assoc.

[38] Coupland C, Hill T, Morriss R (2016). Antidepressant use and risk of cardiovascular outcomes in people aged 20 to 64: cohort study using primary care database. BMJ.

[39] Pizzi C, Rutjes AWS, Costa GM (2011). Meta-analysis of selective serotonin reuptake inhibitors in patients with depression and coronary heart disease. Am J Cardiol.

[40] Stewart JC, Perkins AJ, Callahan CM (2014). Effect of collaborative care for depression on risk of cardiovascular events: data from the IMPACT randomized controlled trial. Psychosom Med.

[41] Kimmel SE, Schelleman H, Berlin JA (2011). The effect of selective serotonin re-uptake inhibitors on the risk of myocardial infarction in a cohort of patients with depression. Br J Clin Pharmacol.

[42] Coupland C, Dhiman P, Morriss R (2011). Antidepressant use and risk of adverse outcomes in older people: population based cohort study. BMJ.

[43] Biffi A, Scotti L, Corrao G (2017). Use of antidepressants and the risk of cardiovascular and cerebrovascular disease: a meta-analysis of observational studies. Eur J Clin Pharmacol.

[44] Rieckmann N, Kronish IM, Shapiro PA (2013). Serotonin reuptake inhibitor use, depression, and long-term outcomes after an acute coronary syndrome: a prospective cohort study. JAMA Intern Med.

[45] Hayashi T, Sumi D, Matsui-Hirai H (2003). Sarpogrelate HCl, a selective 5-HT2A antagonist, retards the progression of atherosclerosis through a novel mechanism. Atherosclerosis.

[46] Ren S, Qian S, Wang W (2013). Prospective study of sarpogrelate hydrochloride on patients with arteriosclerosis obliterans. Ann Thorac Cardiovasc Surg Off J Assoc Thorac Cardiovasc Surg Asia.

[47] Takahara M, Kaneto H, Katakami N (2014). Effect of sarpogrelate treatment on the prognosis after endovascular therapy for critical limb ischemia. Heart Vessels.

[48] Lee D-H, Chun EJ, Hur JH (2017). Effect of sarpogrelate, a selective 5-HT2A receptor antagonist, on characteristics of coronary artery disease in patients with type 2 diabetes. Atherosclerosis.

[49] Rami M, Guillamat-Prats R, Rinne P (2018). Chronic intake of the selective serotonin reuptake inhibitor fluoxetine enhances atherosclerosis. Arterioscler Thromb Vasc Biol.

[50] Sauer WH, Berlin JA, Kimmel SE (2003). Effect of antidepressants and their relative affinity for the serotonin transporter on the risk of myocardial infarction. Circulation.

[51] Kim Y, Lee YS, Kim MG (2019). The effect of selective serotonin reuptake inhibitors on major adverse cardiovascular events: a meta-analysis of randomized-controlled studies in depression. Int Clin Psychopharmacol.

[52] Schlienger RG, Fischer LM, Jick H, Meier CR (2004). Current use of selective serotonin reuptake inhibitors and risk of acute myocardial infarction. Drug Saf.

[53] Fumeron F, Betoulle D, Nicaud V (2002). Serotonin transporter gene polymorphism and myocardial infarction: etude cas-témoins de l’infarctus du myocarde (ECTIM). Circulation.

[54] Doggrell SA (2003). The role of 5-HT on the cardiovascular and renal systems and the clinical potential of 5-HT modulation. Expert Opin Investig Drugs.

[55] Shimizu Y, Minatoguchi S, Hashimoto K (2002). The role of serotonin in ischemic cellular damage and the infarct size-reducing effect of sarpogrelate, a 5-hydroxytryptamine-2 receptor blocker, in rabbit hearts. J Am Coll Cardiol.

[56] Simpson PJ, Schelm JA, Jakubowski JA, Smallwood JK (1991). The role of serotonin (5HT2) receptor blockade in myocardial reperfusion injury: effects of LY53857 in a canine model of myocardial infarction. J Pharmacol Exp Ther.

[57] Yang BC, Virmani R, Nichols WW, Mehta JL (1993). Platelets protect against myocardial dysfunction and injury induced by ischemia and reperfusion in isolated rat hearts. Circ Res.

[58] Grover GJ, Sargent CA, Dzwonczyk S (1993). Protective effect of serotonin (5-HT2) receptor antagonists in ischemic rat hearts. J Cardiovasc Pharmacol.

[59] Hohlfeld T, Braun M, Strobach H, Schrör K (1994). Protection of reperfused ischemic pig myocardium by nexopamil, a new combined Ca2 + and serotonin antagonist. J Cardiovasc Pharmacol.

[60] Brattelid T, Qvigstad E, Moltzau LR (2012). The cardiac ventricular 5-HT4 receptor is functional in late foetal development and is reactivated in heart failure. PLoS ONE.

[61] Levy FO, Qvigstad E, Krobert KA (2008). Effects of serotonin in failing cardiac ventricle: signalling mechanisms and potential therapeutic implications. Neuropharmacology.

[62] Birkeland JAK, Swift F, Tovsrud N (2007). Serotonin increases L-type Ca2 + current and SR Ca2 + content through 5-HT4 receptors in failing rat ventricular cardiomyocytes. Am J Physiol Heart Circ Physiol.

[63] Birkeland J, Sjaastad K, Brattelid I (2007). Effects of treatment with a 5-HT4 receptor antagonist in heart failure. Br J Pharmacol.

[64] Kjekshus JK, Torp-Pedersen C, Gullestad L (2009). Effect of piboserod, a 5-HT4 serotonin receptor antagonist, on left ventricular function in patients with symptomatic heart failure. Eur J Heart Fail.

[65] Brenner B, Harney JT, Ahmed BA (2007). Plasma serotonin levels and the platelet serotonin transporter. J Neurochem.

[66] Biondi ML, Agostoni A, Marasini B (1986). Serotonin levels in hypertension. J Hypertens Suppl Off J Int Soc Hypertens.

[67] Minuz P, Patrignani P, Gaino S (1979). (2004) Determinants of platelet activation in human essential hypertension. Hypertens Dallas Tex.

